# Arabidopsis JMJD5/JMJ30 Acts Independently of LUX ARRHYTHMO Within the Plant Circadian Clock to Enable Temperature Compensation

**DOI:** 10.3389/fpls.2019.00057

**Published:** 2019-02-01

**Authors:** Matthew A. Jones, Kengo Morohashi, Erich Grotewold, Stacey L. Harmer

**Affiliations:** ^1^School of Biological Sciences, University of Essex, Colchester, United Kingdom; ^2^Department of Plant Biology, University of California, Davis, Davis, CA, United States; ^3^Department of Applied Biological Science, Tokyo University of Science, Noda, Japan; ^4^Department of Biochemistry and Molecular Biology, Michigan State University, East Lansing, MI, United States

**Keywords:** circadian, JMJD5, JMJ30, Arabidopsis, temperature compensation

## Abstract

The circadian system ensures that plants respond appropriately to environmental change by predicting regular transitions that occur during diel cycles. In order to be most useful, the circadian system needs to be compensated against daily and seasonal changes in temperature that would otherwise alter the pace of this biological oscillator. We demonstrate that an evening-phased protein, the putative histone demethylase JMJD5, contributes to temperature compensation. *JMJD5* is co-expressed with components of the Evening Complex, an agglomeration of proteins including EARLY FLOWERING3 (ELF3), ELF4, and LUX ARRHYTHYMO (LUX), which also integrates temperature changes into the molecular clockwork. One role of the Evening Complex is to regulate expression of *PSEUDORESPONSE REGULATOR9* (*PRR9*) and *PRR7*, important components of the temperature compensation mechanism. Surprisingly we find that LUX, but not other Evening Complex components, is dispensable for clock function at low temperatures. Further genetic analysis suggests JMJD5 acts in a parallel pathway to LUX within the circadian system. Although an intact JMJD5 catalytic domain is required for its function within the clock, our findings suggest JMJD5 does not directly regulate H3K36 methylation at circadian loci. Such data refine our understanding of how JMDJ5 acts within the Arabidopsis circadian system.

## Introduction

Light and temperature vary dramatically yet predictably over the course of a diel cycle. In order to anticipate these regular environmental changes, plants have evolved an endogenous oscillator known as the circadian clock. This molecular timing mechanism is entrained by regular changes in light or temperature, but circadian clock pace is compensated against temperature fluctuations, allowing the clock to provide a reliable internal timing reference against which daylength can be measured (Hsu and Harmer, 2014). Circadian timing plays a key role in plant development by allowing developmental transitions, such as flowering time, to be regulated by daylength, as well as permitting anticipation of dawn and dusk (Song et al., 2015; Millar, 2016).

Key components of the plant clock include members of partially redundant transcription factor families that interact via multiple feedback loops (Hsu and Harmer, 2014). A succession of transcription factors (including TIMING OF CAB1 EXPRESSION1 [TOC1], PSEUDORESPONSE REGULATOR [PRR] proteins, CIRCADIAN CLOCK ASSOCIATED1 [CCA1], and LATE ELONGATED HYPOCOTYL [LHY]) negatively regulate gene expression throughout the day and night, comprising a molecular timekeeper that oscillates with an approximate 24-h rhythm (Wang and Tobin, 1998; Alabadi et al., 2001; Nakamichi et al., 2010). Additional regulation is provided by the Evening Complex, a complex of three proteins that repress gene expression in the early portion of the night (Nusinow et al., 2011; Huang et al., 2016). Rare activators of circadian gene expression include REVEILLE (RVE), NIGHT LIGHT-INDUCIBLE (LNK) and LIGHT-REGULATED WD (LWD) proteins (Farinas and Más, 2011; Rawat et al., 2011; Rugnone et al., 2013; Xie et al., 2014; Wu et al., 2016). These transcription factors control each other’s expression via interlinked feedback loops that provide robustness in the face of environmental challenges and generate high-amplitude circadian oscillations (Shalit-Kaneh et al., 2018).

One of the key abiotic challenges facing plant circadian systems is the seasonal variation in ambient temperature, which would accelerate or slow the biological oscillator in the absence of compensatory mechanisms (Bodenstein et al., 2012). Temperature compensation in plants arises from a variety of modifications including changes in transcript accumulation, alternative splicing, and post-translational modifications (Gould et al., 2006; Portolés and Más, 2010; Salomé et al., 2010; Nagel et al., 2014; Filichkin et al., 2015; Marshall et al., 2016; Cha et al., 2017; Hansen et al., 2017). The accumulation of many circadian transcripts is rapidly altered in response to temperature changes (Mizuno et al., 2014b), suggesting various mechanisms for temperature compensation. Interestingly, components of the Evening Complex are necessary to integrate temperature into the circadian system (Mizuno et al., 2014b). The Evening Complex regulates expression of *PRR9* and *PRR7*, providing a mechanism through which clock pace could be maintained across a range of physiologically relevant temperatures (Salomé et al., 2010; Mizuno et al., 2014b). The response of clock components to temperature changes also has important consequences for plant survival. For instance, a component of the Evening Complex, *LUX ARRHYTHMO* (*LUX*), is induced by expression of a cold-inducible transcriptional activator and is necessary for the adoption of freezing tolerance (Chow et al., 2014).

Although an extensive list of circadian transcriptional regulators has been assembled, the molecular mechanisms underlying the function of these proteins are still being elucidated. LNK proteins recruit the basal transcriptional machinery to circadian loci (Ma et al., 2018), whereas PRR proteins form complexes with histone deacetylases to repress gene expression as part of an ordered transition between histone modification states (Malapeira et al., 2012; Wang et al., 2013). We have previously reported the role of JMJD5/JMJ30, a putative histone demethylase, as a conserved component of the circadian system (Jones et al., 2010; Jones and Harmer, 2011; Lu et al., 2011). JMJD5 is co-expressed with the core clock component *TOC1*, and like *toc1* mutants, *jmjd5* mutants have a short period phenotype (Jones et al., 2010; Lu et al., 2011). Unusually for circadian clock mutants, over-expression of JMJD5 delays flowering time despite causing a shortened circadian period (Lu et al., 2011). Subsequent investigation revealed that JMJD5 acts to delay flowering by regulating expression of *FLOWERING LOCUS C* (*FLC*) (Gan et al., 2014). JMJD5 binds to the *FLC* promoter, and *JMJD5* over-expression leads to a reduction in the H3K27me3 repressive mark at this locus and increased *FLC* expression (Gan et al., 2014). JMJD5 has therefore been suggested as a good candidate for altering histone marks so as to modulate circadian-regulated gene expression.

Interestingly, increased ambient temperatures promote accumulation of JMJD5 mRNA and protein (Gan et al., 2014) and so we were curious whether JMJD5 preferentially controls circadian clock pace at higher temperatures. Indeed, our studies show that *jmjd5* mutants have exaggerated clock phenotypes at elevated temperatures and thus temperature compensation is impaired in these plants. We also found that JMJD5 tends to be associated with the *PRR7* promoter and that expression of *PRR7* is altered in *jmjd5* mutants. However, we observe no differences in H3K36 methylation at the *PRR7, PRR9*, or *CCA1* promoters. We also demonstrate that JMDJ5 acts additively with components of the evening complex, known regulators of *PRR7* and *PRR9* (Huang and Nusinow, 2016), and that *lux* seedlings have a temperature-dependent phenotype. These data reveal a role for JMJD5 in circadian temperature compensation.

## Materials and Methods

### Plant Material and Growth Conditions

*jmjd5-1, jmjd5-2*, and *lux-2* seed have previously been described (Hazen et al., 2005; Jones et al., 2010). *jmjd5-1 lux-2* lines were generated by crossing these parental lines. JMJD5-OX lines were generated as follows. The coding sequence of JMJD5 was amplified by PCR and cloned into pENTR and later mobilized into pGWB5 to construct a binary vector, *35S::JMJD5:GFP*. *35S::JMJD5:GFP* lines were transformed into Col-0 plants before selection on MS media with 50 μg/ml kanamycin. All plants were grown at 22°C, under a photoperiod of 12 h-light and 12 h-dark condition. *JMJD5::JMJD5:GFP* lines have previously been described (Jones et al., 2010). *JMJD5::JMJD5(H326A):GFP* lines were generated by using the Quikchange site-directed mutagenesis kit (Stratagene, La Jolla, CA, United States) to introduce a single amino acid substitution into pENTR AtJMJD5 (Jones et al., 2010). This mutated construct was then used in conjunction with pGWB4 (Nakagawa et al., 2007) to generate pGWB4 AtJMJD5(H326A). pGWB4 AtJMJD5(H326A) was moved into Agrobacterium strain GV3101 and transformed into *jmjd5-1 CCR2::LUC* plants by Agrobacterium-mediated transformation (Clough and Bent, 1998). Transformants were selected on Murashige–Skoog media supplemented with 3% (wt/vol) sucrose containing 50 μg/mL hygromycin (EMD Chemicals).

### Chromatin Immunoprecipitation

Seedlings were grown on 0.5x MS media for 10 days under 12:12 light:dark cycles. ChIP experiments were performed as previously described using anti-GFP (ab290; abcam), anti-histone H3K36me1 (ab9048; Abcam), H3K36me2 (ab9049; Abcam), and H3K36me3 (ab9050; Abcam) respectively (Morohashi et al., 2007, 2009). Raw data from four biological replicates were normalized to input before being presented relative to controls, as previously reported (Bolduc et al., 2012; Eveland et al., 2014). Statistical significance was assessed using the R statistical environment (R Core Team, 2018).

### Immunoblotting

For each time point, approximately 30 seedlings were collected, frozen in liquid nitrogen and stored at -80°C until analysis. Plant tissue was ground in homogenization buffer (25 mM MOPS (pH 7.8), 0.25 M sucrose, 0.1 mM MgCl_2_, Complete EDTA-free protease-inhibitor cocktail (Roche) at 4°C. Protein concentrations of total cell extracts were then determined by Bradford assay (Bio-rad). 50 μg of each sample was analyzed by immunoblotting, using anti-GFP antibody (ab290; Abcam), anti-H3K4me3 (ab8580; Abcam), anti-H3K27me2 (ab24684; Abcam), anti-H3K36me3 (ab9050; Abcam), anti-H3K79me3 (ab2621; Abcam) anti-H3 carboxyl terminus (ab1791; Abcam), or anti-UGPase antibody (AS05086, AgriSera) followed by a secondary antibody, goat anti-rabbit IgG-HRP (1858415, Pierce). ECL Plus reagent (GE Healthcare) was used to generate chemiluminescence which was then detected with BioMax Light Film (Kodak). Presented data is representative of three biological replicates.

### Luciferase Imaging

To complete luciferase imaging individual seedlings were entrained for 6 days in 12:12 light:dark cycles under white light on half-strength MS media with 3% supplemental sucrose before being sprayed with 3 mM D-luciferin in 0.1% Triton X-100. Plants were then transferred to free-running conditions under 30 μmol m^-2^ s^-1^ red light and 20 μmol m^-2^ s^-1^ blue light, with images being captured every 2 h (Jones et al., 2010, 2012). Patterns of luciferase activity were fitted to cosine waves using Fourier Fast Transform-Non-Linear Least Squares [FFT-NLLS, (Plautz et al., 1997)] to estimate circadian period length. RAE is a measure of rhythmic robustness, with a value of 0 indicating an exact fit to a cosine wave (Plautz et al., 1997). Presented data is representative of three biological replicates.

### qRT-PCR

RNA was isolated and qRT-PCR performed as previously described (Jones et al., 2010). Briefly, total RNA and cDNA synthesis were completed using TRIzol reagent and SuperScript II reverse transcriptase, respectively, following the manufacturer’s protocol (Invitrogen). Real-time qRT-PCR was performed using a BioRad CFX96 Real-Time system. Samples were run in triplicate, with starting quantity estimated from critical thresholds using the standard curve of amplification. Data for each sample were normalized to *PP2a* expression as an internal control and are the average of at least three independent experiments. Primer sets used are described in Supplementary Data Sheet S1.

### Accession Numbers

Sequence data from this article can be found in the Arabidopsis Genome Initiative database under the following accession numbers: *CCA1*, At2g46830; *ELF3*, At2g25930; *ELF4, At2g40080; JMJD5, At3g20810; LUX*, At3g46640; *PP2A*, At1g13320; *PRR7*, At5g02810; *PRR9*, At2g46790.

## Results

### *jmjd5* Seedlings Display Defective Temperature Compensation

**FIGURE 1:**
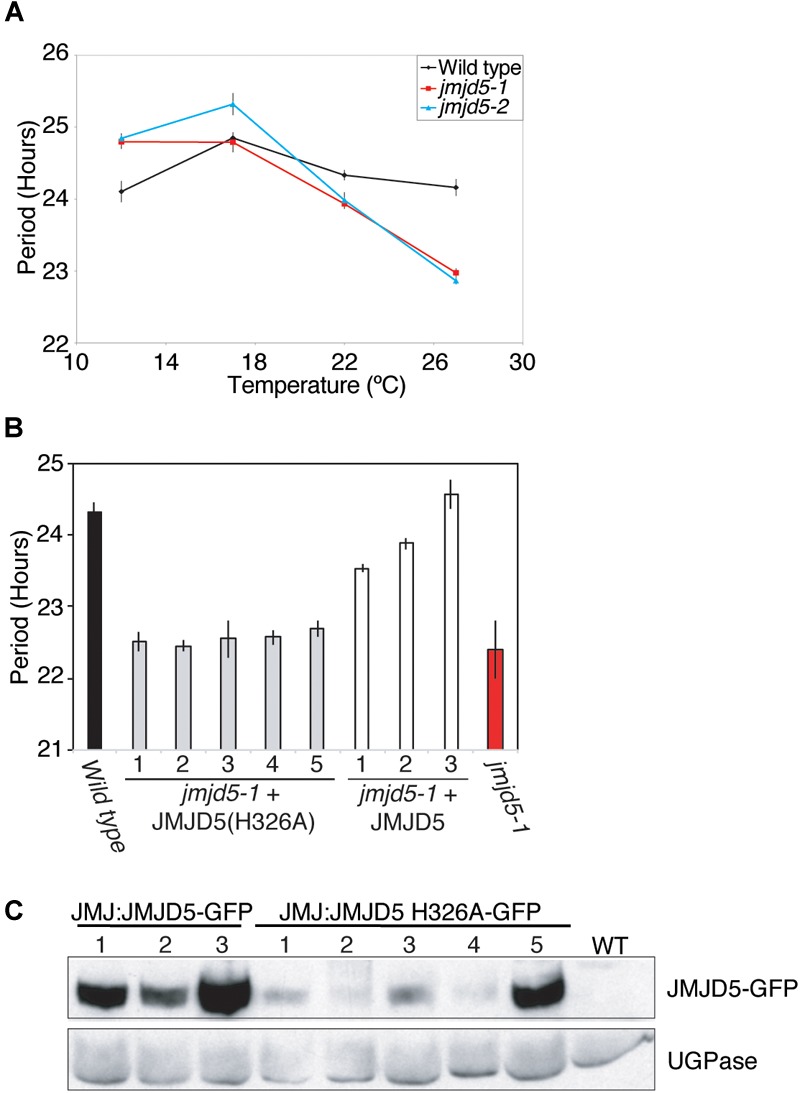
*JMJD5* contributes to temperature compensation. **(A)** Temperature response curve describing period length of wild type, *jmjd5-1* and *jmjd5-2* plants when held at different temperatures under constant conditions. Plants were grown under 60 μmol m^-2^ s^-1^ white light under 12:12 light:dark cycles before being transferred to constant red + blue light (30 μmol m^-2^ s^-1^ and 20 μmol m^-2^ s^-1^ respectively) for 5 days at the indicated temperature. Circadian period estimates of *CCR2::LUC* luminescence were calculated by FFT-NLLS (Plautz et al., 1997). SEM is indicated, n ≥ 20. **(B)** Circadian period estimates of plants transformed with either wild-type *JMJD5* or a mutated *JMJD5* construct bearing a H326A substitution. Seedlings were grown as described in **(A)** before being transferred to constant red + blue light at 27°C. Period estimates from wild type and *jmjd5-1* seedlings are presented for comparison. **(C)** Immunoblot analysis of JMJD5-GFP protein levels at ZT12. *jmjd5-1* plants transformed with *JMJD5JMJD5-GFP* or *JMJD5::JMJD5(H326A)-GFP were* grown under 12:12 LD cycles for 10 days before samples were taken at ZT12. Equal protein loading was assessed using an anti-UGPase antibody (below). All presented data are representative of three independent biological replicates.

We have previously shown that *jmjd5* mutants display increased sensitivity to high levels of monochromatic red light (Jones et al., 2010). To further evaluate the effect of abiotic stimuli on *jmjd5* mutants, we examined clock function in these plants at a range of physiologically relevant temperatures. Although luciferase activity in wild-type plants expressing *CCR2::LUC* has a relatively constant circadian period of approximately 24 h at temperatures between 12 and 27°C [Figure 1A, (Gould et al., 2013)], *jmjd5* mutants display a shorter circadian period at increasing temperatures. As previously reported (Jones et al., 2010; Lu et al., 2011), *jmjd5* mutants have a modest short period phenotype at 22°C (Figure 1A). We found this phenotype to be much more pronounced at 27°C, at which temperature the periods of both *jmjd5-1* and *jmjd5-2* are approximately 1.5 h shorter than those of the wild-type control. Interestingly, at 12°C, both *jmjd5-1* and *jmjd5-2* seedlings have a slightly longer period than wild type (Figure 1A). These data indicate that *jmjd5* mutants are less able than wild type to compensate the clock mechanism against changes in temperature.

### Conserved Residues Within the jmjC Domain Are Required for the Clock Function of JMJD5

Our previous work demonstrated that the human and Arabidopsis homologs of JMJD5 are interchangeable between the circadian systems of these species, suggesting that the biological function of this protein has been conserved (Jones et al., 2010). Human JMJD5 was initially characterized as a histone H3 lysine 36 dimethyl (H3K36me2) demethylase (Hsia et al., 2010), while Arabidopsis JMJD5 has been ascribed H3K27me2 and H3K9me3 demethylase activity (Gan et al., 2014; Lee et al., 2018). More recently, it has been suggested that human JMJD5 is primarily a protein hydroxylase (Del Rizzo et al., 2012; Youn et al., 2012; Wilkins et al., 2018). Both lysine demethylation and protein hydroxylation proceed via a dioxygenase mechanism that requires the co-ordination of Fe(II) and 2-oxoglutarate within the jmjC domain (reviewed in Webb et al., 2009; Mosammaparast and Shi, 2010). In order to determine whether this co-factor co-ordination is necessary for Arabidopsis JMJD5 activity in the clock, we introduced a point mutation (His^326^→Ala, H326A) that removes one of the conserved histidine residues required for Fe(II) binding and that has previously been reported to abrogate the histone demethylase activity of human JMJD5 *in vitro* (Hsia et al., 2010). Expression of this mutant JMJD5(H326A) protein under the control of the *JMJD5* promoter did not rescue the mutant circadian phenotype of *jmjd5-1* seedlings (Figures 1B,C). While these data do not preferentially support a specific hypothesis relating to the biochemical activity of JMJD5, they demonstrate that His^326^ is required for JMJD5 clock function *in vivo*.

### JMJD5 May Associate With the Promoters of *CCA1* and *PRR7*

**FIGURE 2:**
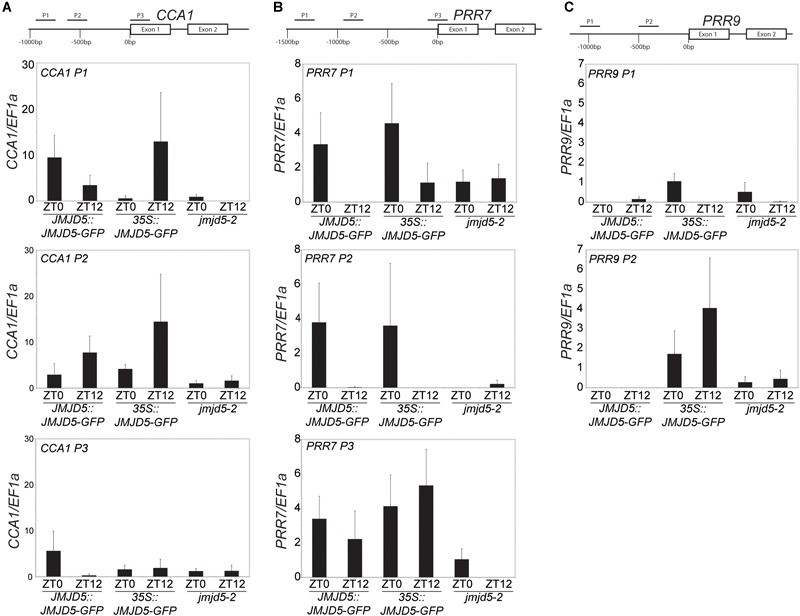
Chromatin Immunoprecipitation assays to evaluate JMJD5-GFP association with circadian gene loci. Enrichment of genomic DNA fragments from the *CCA1* **(A)**, *PRR7* **(B)** and *PRR9* **(C)** promoters following chromatin immunoprecipitation with a GFP antibody. Plants were grown for 10 days at 22°C under 12:12 light:dark cycles and harvested at either ZT0 or ZT12. Presented data represent fold-increases compared to enrichment at the *EF1a* promoter and are the mean of four biological independent replicates. Error bars represent SEM.

To further understand the role of JMJD5 within the circadian system, we used GFP-tagged versions of JMJD5 to determine whether this protein is associated with the promoters of circadian genes. We first examined the promoter of *CCA1*, examining two regions within 1 kb of the transcriptional start site (TSS) and another within the first exon of the *CCA1* gene [Figure 2A, (Dixon et al., 2011)]. We carried out chromatin immunoprecipitation assays with plant tissues harvested either at dusk or dawn (ZT0 or ZT12) using transgenic lines expressing JMJD5-GFP under either its native promoter (*JMJD5:JMJD5-GFP*) or the strong viral *CaMV 35S* promoter (*35S:JMJD5-GFP*). With *JMJD5:JMJD5-GFP* plants, we found JMJD5-GFP to be present at the *CCA1* promoter at both timepoints, but with higher levels of the protein associated with regions P1 and P3 at ZT0 (Figure 2A). Interestingly, the opposite trend was observed at region P2, approximately 500 bp from the transcriptional start site (TSS). Here, JMJD5-GFP association is greater at ZT12 than at ZT0 for both *JMJD5:JMJD5-GFP* and *35S:JMJD5-GFP* lines. Despite these trends, none of these associations were statistically significantly different from *jmjd5-2* seedlings, although JMJD5 association with the CCA1-P1 region at ZT0 approached statistical significance (*p* = 0.096, ANOVA).

*jmjd5* mutants have pronounced period phenotypes at increased temperatures, which is reminiscent of the phenotype of *prr7 prr9* plants [although in these mutants period increases with temperature (Salomé et al., 2010)]. We therefore examined JMJD5 association with regions within the *PRR7* promoter (Figure 2B). We found that JMJD5-GFP tended to be present at the *PRR7* TSS throughout the day, even when the endogenous promoter was used to drive *JMJD5-GFP* expression. In more distal regions such as *PRR7* P1 and P2, however, JMJD5-GFP association is higher at dawn when compared to dusk. Interestingly, this was true both for JMJD5 expressed under the constitutive 35S and the clock-regulated *JMJD5* promoter, suggesting that JMJD5-GFP requires a diurnally cycling partner to associate with these promoter regions, or that JMJD5-GFP is post-transcriptionally regulated. As seen with the *CCA1* promoter region, none of these associations were significantly different from controls, although JMJD5 association with the PRR7-P3 region at ZT12 approached statistical significance (*p* = 0.096, ANOVA). This lack of statistical significance is likely due to variability in our dataset. We also assessed two regions within the *PRR9* promoter (Figure 2C). In contrast to the *PRR7* promoter, we did not detect any association of JMJD5 with these regions when using *JMJD5:JMJD5-GFP* lines. In plants overexpressing JMJD5, we did find association with one portion of the *PRR9* promoter (Figure 2C), but it remains unclear whether this is an artifact of overexpression.

### Over-Expression of JMJD5 Alters Global Patterns of Histone Methylation

**FIGURE 3:**
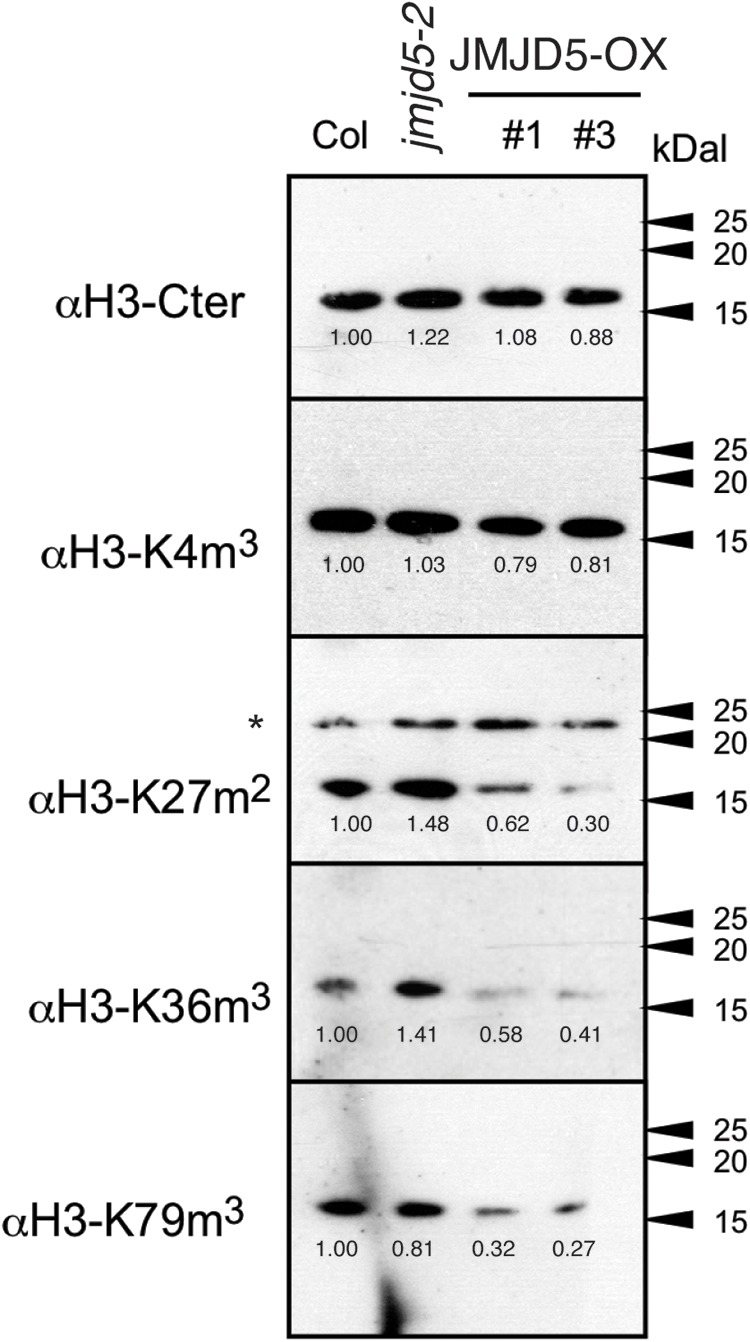
Assessment of global histone methylation in *jmjd5* and *JMJD5* over-expressing lines. Immunoblot analysis of histone H3 methylation in wild type, *jmjd5-2* and transgenic plants expressing *JMJD5* under the control of a *35S* promoter *(35S:JMJD5-GFP Col-0).* Plants were grown at 22°C under constant white light for 2 weeks before harvesting. Blots were incubated with the indicated antibodies. Values are normalized to wild type for each exposure, ^∗^indicates non-specific bands. Presented data are representative of three biological independent replicates.

Overexpression of Arabidopsis JMJD5 has been implicated in both H3K9me3 and H3K27me3 demethylation (Gan et al., 2014; Lee et al., 2018) and so we used specific antibodies to assess whether mis-expression of JMJD5 is sufficient to alter the global accumulation of multiple histone methylation marks (Figure 3). Plants were grown without entrainment for 2 weeks before harvesting to desynchronize circadian rhythms between cells (Guerriero et al., 2012; Gould et al., 2018) and thus mitigate any differences in histone methylation arising from the period phenotypes of *JMJD5* overexpressing and mutant plants (Jones et al., 2010; Lu et al., 2011). While we observed little change in H3K4me3 accumulation in either *jmjd5-2* mutants or *35S:JMJD5-GFP* seedlings, we did observe alterations in overall H3K27me2, H3K36me3 and H3K79me3 modifications when we manipulated expression levels of *JMJD5.* H3K36me3 levels are decreased in *35S:JMJD5-GFP* lines, whereas levels of this mark are modestly increased in *jmjd5-2* plants. We saw similar decreases in H3K27me2 and H3K79me3 methylation in *35S:JMJD5-GFP* plants although there was no apparent change in the abundance of these modified histones in *jmjd5-2* mutants. These data demonstrate that alteration of *JMJD5* expression levels results in genome-wide changes in the levels of some, but not all histone methylation marks.

**FIGURE 4:**
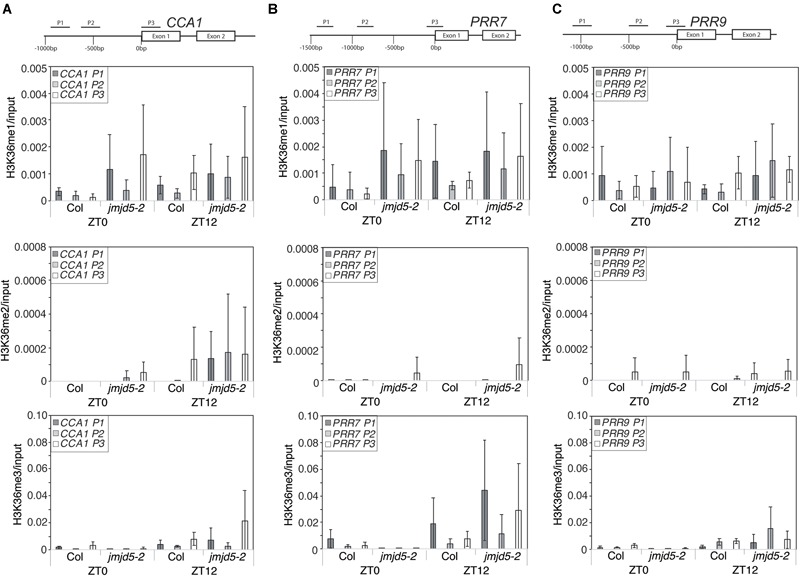
Chromatin Immunoprecipitation assays to evaluate histone H3K36 methylation at circadian gene loci. Enrichment of genomic DNA fragments from the *CCA1* **(A)**, *PRR7* **(B)** and *PRR9* **(C)** promoters following chromatin immunoprecipitation with H3K36me1, H3K36me2 or H3K36me3 antibodies. Plants were grown for 10 days at 22°C under 12:12 light:dark cycles and harvested at either ZT0 or ZT12. Data from four independent experiments is shown and represents fold-increases compared to input. Error bars show standard deviation.

As global H3K36me3 histone methylation is altered in plants lacking or over-expressing *JMJD5* (Figure 3) and JMJD5 has been reported to have H3K36me2 demethylase activity (Hsia et al., 2010) we next examined whether histone H3K36 methylation is altered at circadian loci at either dawn or dusk. We identified little change in H3K36me1 methylation in either wild-type or *jmjd5-2* seedlings at any of the examined positions in *CCA1* (Figure 4A, top panel), but we did observe modest increases in H3K36me2 methylation at the *CCA1* promoter at ZT12 in *jmjd5-2* compared to wild type (Figure 4A, middle panel). Surprisingly, *jmjd5-2* plants did not show a significant enrichment of either H3K36me2 or H3K36me3 marks at *CCA1* P3 compared to wild type despite the higher global levels of H3K36me3 methylation in this mutant (Figure 3, ANOVA, *p* > 0.1). These results suggest that JMJD5 is not functioning simply as a H3K36me2 demethylase.

To further test this hypothesis, we next examined whether H3K36 methylation was altered at the *PRR7* promoter (Figure 4B). We did not detect significant levels of H3K36me1 or H3K36me2 enrichment in either wild-type or *jmjd5*-2 seedlings at the *PRR7* promoter (*p* > 0.1, ANOVA). Once again, *jmjd5-2* seedlings did not show significant enrichment of any H3K36 methylation mark at either time point at this locus (Figure 4B). Finally, we did not observe any significant enrichment of H3K36 methylation at the *PRR9* promoter at either time in any genotype (Figure 4C). It therefore appears that loss of JMJD5 activity does not significantly alter levels of H3K36 methylation at these clock gene loci.

### *jmjd5* Seedlings Have Altered Expression Levels of Clock Genes at 27°C

**FIGURE 5:**
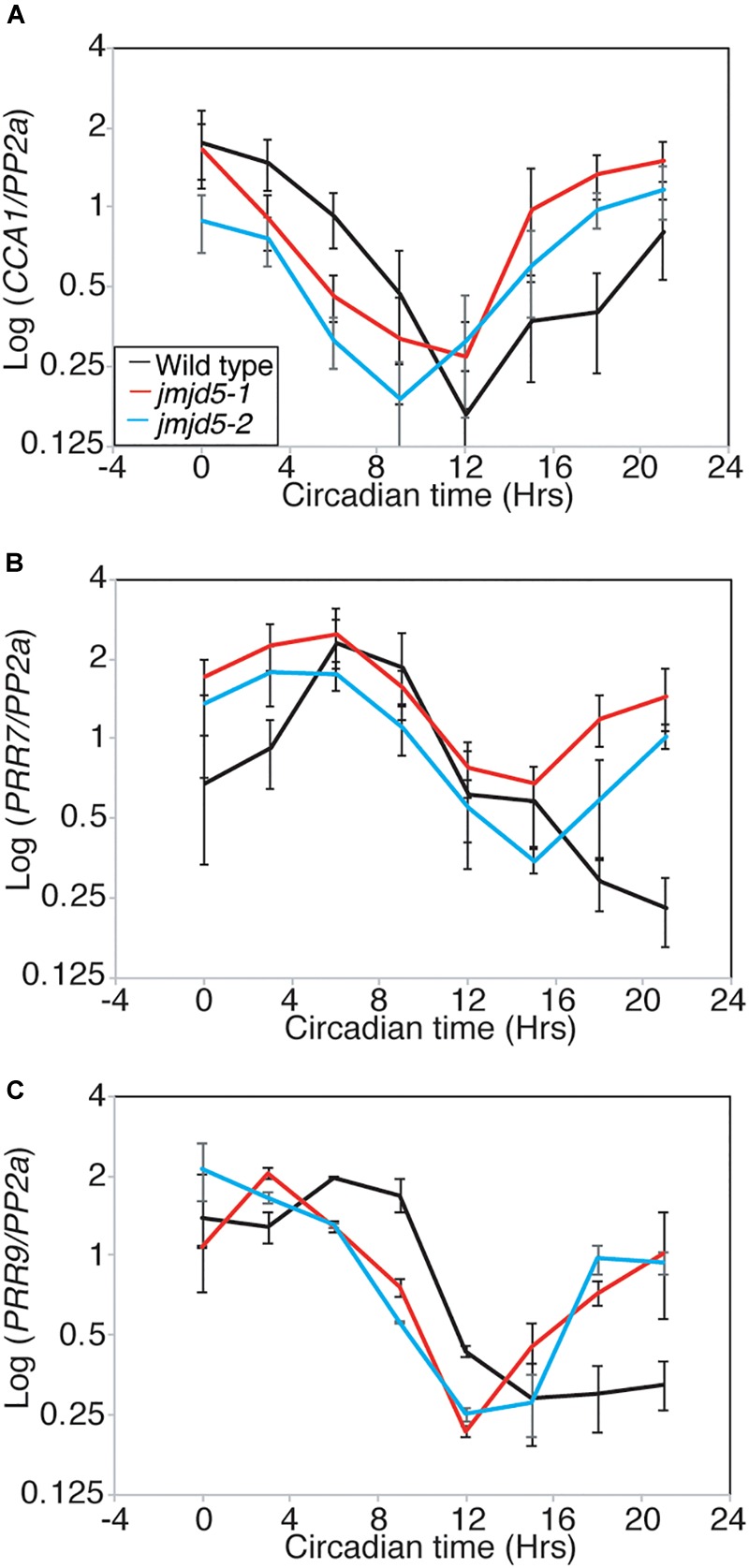
Relative accumulation of circadian transcripts in *jmjd5* mutants under constant light at 27°C. Transcript accumulation in wild type (Col, black line), *jmjd5-1* (red), and *jmjd5-2* (blue) mutants was compared using qRT-PCR. Levels of *CCA 1* **(A)**, *PRR7* **(B)**, and *PRR9* **(C)** were assessed. Plants were entrained to 12:12 LD cycles for 6 d before being moved to constant conditions with 60 μmol m^-2^ s^-1^ white light at 27°C. mRNA levels for each gene were normalized to *PP2a.* Data are the mean of four independent biological replicates; SEM is shown.

We have previously shown that *jmjd5* seedlings display reduced peak levels of *CCA1* and *LHY* transcript when maintained in 120 μmol^-1^ s^-1^ monochromatic red light (Jones et al., 2010). Given our observations that *jmjd5* mutant plants have a more pronounced period phenotype at 27°C (Figure 1A), and that JMJD5-GFP may bind to the promoters of *CCA1* and *PRR7* (Figure 2), we were curious whether expression of *CCA1* and *PRR7* was altered at high temperatures. We grew plants in 12:12 light:dark cycles at 22°C for 10 days before transfer at dawn to constant white light (60 μmol^-1^ s^-1^) and 27°C, thereafter harvesting tissue every 3 h as previously described (Jones et al., 2010). Consistent with the short circadian period observed in *jmjd5* mutants under these conditions, the phases of both *CCA1* and *PRR9* peak transcript accumulation are early compared to wild-type controls (Figures 5A,C). However, peak levels of both of these transcripts are similar to wild type, suggesting that *jmjd5* deficiency leads to a short period phenotype via different mechanisms under either monochromatic red light or high temperature. Interestingly, *PRR7* transcript levels are elevated throughout the circadian cycle, with the troughs of *PRR7* transcript accumulation in *jmjd5-1* being approximately twice that of the wild-type (Figure 5B). Indeed, *PRR7* transcript levels are higher in *jmjd5* plants at all times except at the peaks, at which point they are indistinguishable from wild type (Figure 5B). In contrast, no consistent changes in *PRR9* levels were observed in the *jmjd5* mutants (Figure 5C), consistent with the greater enrichment of *PRR7* than *PRR9* loci in JMJD5 chromatin immunoprecipitation experiments (Figure 2). These data suggest that JMJD5 acts to repress expression of *PRR7*.

### JMJD5 Acts in Parallel With LUX to Alter Expression of *PRR7*

**FIGURE 6:**
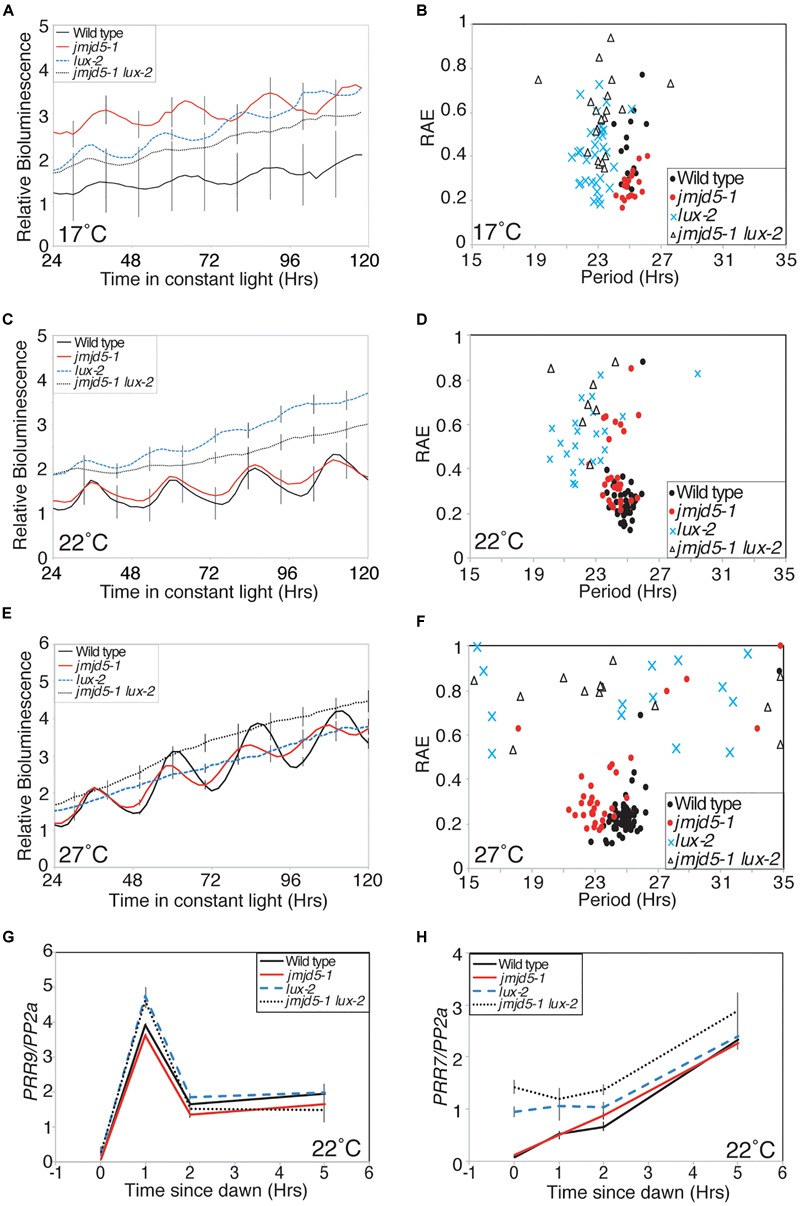
Genetic interaction between *JMJD5* and *LUX.* **(A–F)** Bioluminescence of seedlings containing a *CCR2:LUC* reporter construct. Wild type (Col, black line), *jmdj5-1* (red), *lux-2* (blue) and *jmjd5-1 lux-2* (dotted) plants were entrained to 12:12 LD cycles for 6 days before being moved to constant conditions at either 17°C **(A,B)**, 22°C **(C,D)**, or 27°C **(E,F)** under red+blue light (30 μmol m^-2^ s^-1^ and 20 μmol m^-2^ s^-1^ respectively). Error bars indicate the standard error of the mean (SEM) and are displayed every 10 h for clarity, *n* ≥ 20. Presented data are representative of three independent replicates. **(G,H)** *PRR9* **(G)** and *PRR7* **(H)** transcript accumulation from dawn. Wild type, *jmjd5-1, lux-2*, and *jmjd5-1 lux-2* seedlings were grown under 12:12 light:dark cycles for 10 days before samples were harvested at the indicated times. Level of transcripts of interest was normalized to *PP2a* and is the mean of four independent biological replicates; SEM is shown.

Recent advances have suggested that many evening phased proteins agglomerate into an Evening Complex that acts to repress expression of target genes (Nusinow et al., 2011; Huang et al., 2016). Mutants of Evening Complex components, including *lux, elf3* and *elf4*, are arrhythmic when held under constant light at 22°C (Hicks et al., 1996; Doyle et al., 2002; Hazen et al., 2005). Since JMJD5 is also an evening phased clock component, and *jmjd5* mutants have a more pronounced phenotype at increased temperatures (Figure 1A), we investigated whether the phenotype of Evening Complex mutants was rescued at lower temperatures. We grew plants under 12:12 diel cycles for 6 days before releasing plants into constant red+blue light at 17, 22, or 27°C (Figures 6A–F). The rhymicity of *lux-2* lines decreased as temperature increased. At 17°C *lux-2* mutants are rhythmic, although they display a significantly shorter circadian period (22.84 h ± 0.14) than wild-type (25.11 h ± 0.12). In contrast, *elf4-1* and *elf3-1* mutants remain arrhythmic at this lower temperature (Supplementary Figure S1). At 27°C, *lux-2* seedlings are completely arrhythmic, with only 8% of seedlings returning a rhythm that met our quality threshold (RAE score < 0.6), compared to 58% of seedlings scored as rhythmic at 22°C and 84% of seedlings at 17°C, respectively.

We have previously shown that *jmjd5 toc1* double mutants have an additive circadian phenotype, suggesting that JMJD5 and TOC1 act within the circadian system via distinct pathways despite the similar phenotypes of the single mutants and their co-regulated expression (Jones et al., 2010; Jones and Harmer, 2011). To determine the genetic relationship between *jmjd5* and *lux*, we examined the circadian phenotype of *jmjd5-1 lux-2* seedlings held at 17°C. Interestingly *jmjd5-1 lux-2* mutants become arrhythmic toward the end of the time course (Figures 6A,B) whereas *jmjd5-1* and *lux-2* seedlings display robust rhythmicity throughout the experiment. This exacerbated phenotype in the double mutant suggests that the function of these proteins is at least in part independent of each other.

LUX has previously been shown to repress expression of *PRR9* and *PRR7* by directly binding to their promoters (Helfer et al., 2011; Mizuno et al., 2014b). To further investigate the function of JMJD5 in conjunction with LUX we examined changes in expression of *PRR7* and *PRR9* in *jmjd5-1, lux-2* and *jmjd5-1 lux-2* mutants shortly after dawn under diel cycles at 22°C (Figures 6G,H). *PRR9* expression levels are not appreciably different between wild-type and *jmjd5-1* mutants (Figure 6G), consistent with the lack of enrichment of the *PRR9* promoter in our JMJD5 chromatin immunoprecipitation studies (Figure 2C). In addition, although we observed an increased accumulation of *PRR9* transcripts in *lux-2* plants (similar to that reported by Helfer et al., 2011), we did not see enhanced expression levels in *jmjd5-1 lux-2* mutants. In contrast, we did observe an additive phenotype when we assessed the expression of *PRR7* in *jmjd5 lux* double mutants. Although we saw few differences in *PRR7* expression levels in *jmjd5-1* seedlings compared to wild type controls, *PRR7* expression levels are increased during the night in *lux-2* plants (Figure 6H). We observed a further increase in *PRR7* levels in *jmjd5-1 lux-2* plants compared to *lux-2* in the early morning, from ZT0 until ZT2 (Figure 6H). We therefore propose that JMJD5 acts to repress expression of *PRR7* in parallel with LUX activity, but that it has a negligible role in the control of *PRR9* expression.

## Discussion

### *lux* Mutants Have a Temperature-Dependent Phenotype

The Evening Complex accumulates during the early evening and acts to repress gene expression (Nusinow et al., 2011; Huang and Nusinow, 2016). Recent work has demonstrated that the Evening Complex serves as a signaling hub linking circadian timing with light signaling components, and that the Evening Complex is also required for changes in transcript accumulation in response to temperature (Mizuno et al., 2014a,b; Box et al., 2015; Huang et al., 2016; Ezer et al., 2017). Our work demonstrates that *lux* but not *elf3* or *elf4* mutants have a temperature-dependent effect on circadian function, with *lux* mutants displaying appreciable rhythms at low but not high temperatures (Figure 6A and Supplementary Figure S1). *LUX* transcript levels are decreased immediately after transfer to 16°C, although chromatin immunoprecipitation data suggest the Evening Complex targets many more loci at 17°C than at 22°C (Mizuno et al., 2014b; Ezer et al., 2017). These data suggest that the Evening Complex is reconfigured at these lower temperatures. One possible explanation for enhanced rhythmicity in *lux* relative to *elf3* and *elf4* mutants at low temperatures might be a temperature-dependent function of the *LUX* homolog *BROTHER OF LUX ARRHYTHMO* (Dai et al., 2011).

Although our data indicate LUX activity is dispensable for clock function at low temperatures, LUX is important for other physiological responses of plants to cold. *LUX* transcript accumulation remains rhythmic for multiple days after transfer to 4°C and *LUX* is necessary for the acquisition of freezing tolerance (Bieniawska et al., 2008; Chow et al., 2014). These latter reports may reflect the induction of *CBF1* expression at 4°C, which has been shown to promote *LUX* transcript accumulation (Gilmour et al., 1998; Chow et al., 2014).

### JMJD5 Has a Role in Temperature Compensation

Interestingly, mutation of *JMJD5* also has temperature-dependent effects, with the mutant phenotype more apparent at 27°C than 22°C, although circadian rhythms are maintained at all temperatures in contrast to the apparent loss of rhythms in *lux, elf3*, and *elf4* lines at 22°C (Figures 1A, 5 and Supplementary Figure S1). Despite these results, it is important to note that JMJD5 and Evening Complex activities do not completely overlap. We observed increased accumulation of *PRR7* transcript in *jmjd5-1 lux-2* plants grown under diel cycles as compared to either single mutant, but observed no difference in *PRR9* accumulation under these conditions (Figures 6B,C). Similarly, JMJD5 was only found to associate with the *PRR9* locus when it was over-expressed, unlike LUX and ELF3 (Figure 2C, Dixon et al., 2011; Helfer et al., 2011; Chow et al., 2012; Ezer et al., 2017). Such results are similar to our previous reports indicating that JMJD5 and TOC1 [another Evening Complex component (Huang et al., 2016)] have distinct roles within the circadian system (Jones et al., 2010; Jones and Harmer, 2011), and support a model whereby JMJD5 acts in concert with the Evening Complex to modulate expression of a subset of target genes. Remarkably, JMJD5 accumulation is enhanced at elevated temperatures so that JMJD5 is present at increased levels throughout the night (Gan et al., 2014). It is therefore possible that high temperatures increase the prevalence of JMJD5 at the *PRR7* promoter, leading to transcriptional repression. As the redundancy of the plant circadian system has recently been ascribed to protecting against environmental perturbation (Shalit-Kaneh et al., 2018), it is possible that increased *PRR7* expression in *jmjd5* mutants contributes to compromised circadian function.

### JMJD5 Does Not Substantially Alter Histone Methylation at Circadian Loci

Although the loss of JMJD5 shortens circadian period in humans, plants, and flies (Jones et al., 2010; Lu et al., 2011; Shalaby et al., 2018), the biochemical role of human JMJD5 remains controversial, with histone demethylase and protein hydroxylase activities reported (Hsia et al., 2010; Del Rizzo et al., 2012; Youn et al., 2012; Wilkins et al., 2018). The plant homolog of JMJD5 has been ascribed H3K27me2 and H3K9me3 demethylase activity, although these activities have not been directly demonstrated *in vivo* (Gan et al., 2014; Lee et al., 2018). Our data shows that global levels of H3K36me3 and H3K79me3 marks are also altered by mutation or over-expression of JMJD5 (Figure 3). Given the broad range of histone modifications induced by JMJD5 mis-expression (Figure 3, Gan et al., 2014; Lee et al., 2018), and the lack of significant changes in H3K36 methylation in the promoters investigated (Figure 4), it seems more likely that JMJD5 acts indirectly to alter histone methylation rather than acting as a canonical histone demethylase. Whether specific chromatin marks act as regulators of gene expression or instead are primarily a consequence of transcription factor-mediated control of gene expression is debated. Studies demonstrating correlations between transcription factor binding, histone marks, and gene expression (Benveniste et al., 2014; Ahsendorf et al., 2017) do not distinguish between direct effects of histone modifications on gene regulation and the indirect recruitment of histone modifying enzymes by transcriptional regulators. The finding that daily rhythms in peak levels of H3K4 and H3K36 tri-methylation at clock-regulated promoters in mouse liver lag peak levels of gene expression (Le Martelot et al., 2012) argue that for at least some clock loci changes in these histone marks are not playing a causal role in daily rhythms in gene expression. Since the human JMJD5 protein has been reported to hydroxylate a transcription factor, a protein involved in chromatin stability, and a protein involved in translation (Youn et al., 2012; Wilkins et al., 2018), it is possible that the global changes in gene expression observed in plants mis-expressing JMJD5 (Gan et al., 2014) are caused by changes in protein hydroxylation. In this scenario, the changes in global histone marks we observe in *jmjd5* mutants (Figure 3) might be indirectly caused by changes in gene expression. Although the specificity and substrates of JMDJ5 remain elusive, an intact JMJD5 catalytic domain is required to rescue the circadian phenotype of *jmjd5* lines (Figures 1B,C). We therefore propose that JMJD5 acts to post-translationally modify protein function to regulate the circadian system in response to temperature changes, and that JMJD5 acts alongside LUX and other Evening Complex proteins to maintain robust circadian rhythms in a variety of environmental conditions.

## Note Added in Proof

During review of this manuscript, Saran et al. (2018) found that mouse JMJD5 facilitates both proteasomal degradation of CRY1, and CRY1-mediated repression of clock gene expression.

## Author Contributions

MJ, KM, EG, and SH designed the experiments and analyzed the data. MJ and KM performed the experiments while MJ and SH wrote the manuscript.

## Conflict of Interest Statement

The authors declare that the research was conducted in the absence of any commercial or financial relationships that could be construed as a potential conflict of interest.
