# Supplementary material for: Arabidopsis JMJD5/JMJ30 Acts Independently of LUX ARRHYTHMO Within the Plant Circadian Clock to Enable Temperature Compensation
Source: Front Plant Sci. 2019 Feb 1;10:57. doi: 10.3389/fpls.2019.00057 (PMC6367231; doi:10.3389/fpls.2019.00057)
Supplement: Supplementary file 1 [file Data_Sheet_1.PDF]

ChIP CCA1 P1 F	ACCCTTCATGCATGGTTAGC
ChIP CCA1 P1 R	CATTCTCGTGCGGTTCACTA
ChIP CCA1 P2 F	GTCGACAAACTGGTGGGAGA
ChIP CCA1 P2 R	TCCGGGACTACCTGAAAGG
ChIP CCA1 P3 F	TTCGTCTGGAGAAGATCTGG
ChIP CCA1 P3 R	GTCCACCTTTCACGTTGCTT
ChIP EF1a F	CAGGATTGACCACTGAGGTCAA
ChIP EF1a R	TCACCTGGAAGTGCCTCAAGA
ChIP PRR7 P1 F	GCGTGAAGGAACACTGAAGG
ChIP PRR7 P1 R	ACGACGTTATCACGGAGCTT
ChIP PRR7 P2 F	TGTCGATATGTCCGAGTGGT
ChIP PRR7 P2 R	GGTGGGTAAGGAAAACGTCA
ChIP PRR7 P3 F	GGGTTTATGGCTGTGTTTTGA
ChIP PRR7 P3 R	CTCCGGTCTTTCGATCAGTG
ChIP PRR9 P1 F	GGATCTTTTTCTTCGTCAATGG
ChIP PRR9 P1 R	TGATGTGGACAGTGCGTAAT
ChIP PRR9 P2 F	GCCGCGATACAGAGAAAATC
ChIP PRR9 P2 R	TTCGATCACAACCACGAAA
ChIP PRR9 P3 F	GCCGCGATACAGAGAAAATC
ChIP PRR9 P3 R	TTCGATCACAACCACGAAA
JMJD5 Cloning F	caccATGTCAGGAGCTACCACCGCTTCC
JMJD5 Cloning R	CGAGCTAGAAGATTCTGCTTCATTGC
qPCR CCA1 F	TAACGTGGCCAAAATGATGC
qPCR CCA1 R	GTTCTCCACAACCGATTGGT
qPCR JMJD5 F	GTGGAGGTGGGGAAAAAC
qPCR JMJD5 R	CTGATCAAACAAAGGATGCTG
qPCR PP2a F	TAACGTGGCCAAAATGATGC
qPCR PP2a R	GTTCTCCACAACCGATTGGT
qPCR PRR7 F	GAATGTGCTGAGGCGTTCAGA
qPCR PRR7 R	GGCTGGATTATACCTTGAGAAAGC
qPCR PRR9 F	GTTGAAGAGGAAAGATCGATGCTT
qPCR PRR9 R	CTGCTCTGGTACCGAACCTTTT

Supplemental Table 1. Oligos used in this study
