# Supplementary figures and images for: Arabidopsis JMJD5/JMJ30 Acts Independently of LUX ARRHYTHMO Within the Plant Circadian Clock to Enable Temperature Compensation
Source: Front Plant Sci. 2019 Feb 1;10:57. doi: 10.3389/fpls.2019.00057 (PMC6367231; doi:10.3389/fpls.2019.00057)

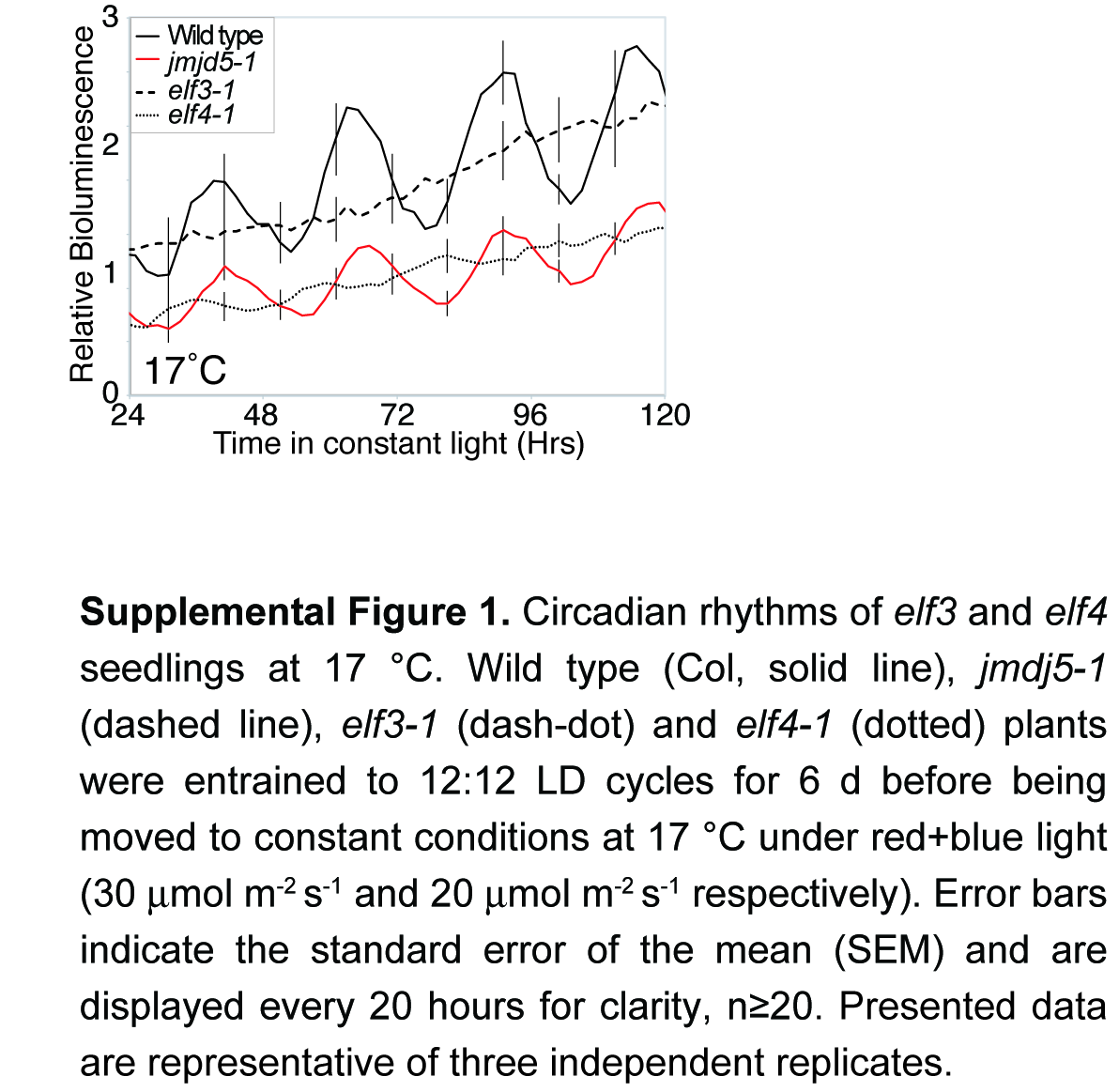

Supplement: Supplementary file 2 [file Image_1.TIF]
